# Antibiotic Resistance in Urinary Pathogens Among Kidney Transplant Recipients: A Persistent Threat

**DOI:** 10.3390/antibiotics14111135

**Published:** 2025-11-10

**Authors:** Büşra Çalışır, Abdullah İbrahim Çalışır, Oktay Rodoplu, Abdulmecit Yıldız, Alparslan Ersoy, Cüneyt Özakın

**Affiliations:** 1 Department of Medical Microbiology, Bursa Uludağ University, Bursa 16059, Turkey; oktayrodoplu@uludag.edu.tr (O.R.); ozakin@uludag.edu.tr (C.Ö.); 2 Division of Nephrology, Department of Internal Medicine, Bursa Uludağ University, Bursa 16059, Turkey; ibrahimcalisir@uludag.edu.tr (A.İ.Ç.); mecit@uludag.edu.tr (A.Y.); alpersoy@uludag.edu.tr (A.E.)

**Keywords:** kidney transplantation, antibiotic resistance, trimethoprim-sulfamethoxazole

## Abstract

**Background:** Urinary tract infections (UTIs) are the most common infections after kidney transplantation and significantly affect patient outcomes. In these immunosuppressed patients, antibiotic resistance is of particular concern due to recurrent infections and limited treatment options. The aim of this study was to evaluate the distribution of microorganisms isolated from urine cultures of kidney transplant patients and to assess their antimicrobial susceptibility patterns. **Methods:** This retrospective study included all adult kidney transplant recipients with positive urine cultures during 2023–2024. Microorganisms were identified using matrix-assisted laser desorption ionization time-of-flight mass spectrometry (MALDI-TOF MS), and antimicrobial susceptibility testing was performed with the Phoenix System according to European Committee on Antimicrobial Susceptibility Testing (EUCAST) guidelines. **Results:** A total of 363 urine samples from 123 patients (85% female) were analyzed. *E. coli* (49%) and *Klebsiella* spp. (24%) were the predominant uropathogens. High resistance rates to ciprofloxacin and trimethoprim–sulfamethoxazole (TMP–SMX) were observed in both species, while carbapenem resistance remained low. Elevated resistance to multiple antibiotics was also detected among *Pseudomonas aeruginosa* and *Acinetobacter* spp., highlighting the importance of continued microbiological surveillance in this population. **Conclusions:** Gram-negative bacteria were the predominant pathogens causing UTIs in kidney transplant recipients. The high resistance rates to ciprofloxacin and TMP–SMX emphasize the need for local antimicrobial surveillance and individualized empirical therapy. Systematic and ongoing monitoring of resistance patterns is essential to optimize infection management in this vulnerable patient group.

## 1. Introduction

Kidney transplantation is regarded as the optimal treatment for patients with end-stage renal disease. Despite the advent of sophisticated surgical techniques, the administration of antimicrobial prophylaxis, and the implementation of hygienic measures, infections persist as a substantial cause of morbidity and mortality in patients who have undergone kidney transplantation. Urinary tract infections (UTIs) are prevalent, with incidence rates ranging from 25% to 75% in the specified patient population, as reported in relevant studies. This wide variation may be attributed to differences in study design, diagnostic criteria (e.g., inclusion of asymptomatic bacteriuria), duration and intensity of post-transplant follow-up, and preventive strategies such as antibiotic prophylaxis and catheter management. It has been determined that approximately 60% of bacteremias in this patient group are caused by urinary tract infection pathogens. This increased susceptibility is multifactorial, reflecting the combined effects of prolonged immunosuppressive therapy, frequent urinary catheterization or ureteral stent use, and anatomical or functional changes in the urinary tract following transplantation [1,2,3]. A study conducted by Chacón-Mora N. et al. reported that an average of 26–43% of kidney transplant recipients experienced at least one urinary tract infection within four years after transplantation [4]. The proper management of UTIs in kidney transplant patients is of paramount importance for both graft and patient survival. The increased utilization of antibiotics for prophylaxis and treatment in transplant recipients has concomitantly led to a rise in infections caused by antibiotic-resistant bacteria. Consequently, the prevalence of bacterial pathogens and antibiotic resistance is a crucial factor in the empirical treatment of such infections [1,2,3,4,5]. The objective of this study is to examine the distribution of microorganisms cultivated in urine cultures and the antimicrobial susceptibility patterns of these microorganisms in patients with a history of kidney transplantation who were admitted to the Nephrology Organ Transplantation Clinic at Bursa Uludağ University in 2023 and 2024.

## 2. Results

In the present study, all adult kidney transplant recipients with positive urine cultures between January 2023 and December 2024 were included. In total, 363 cultures from 123 patients (representing 18% of the total sample) were found to be positive out of 1992 urine cultures from 512 patients admitted to the Nephrology Transplant Service at our hospital. Of the patients with positive urine cultures, 105 (85%) were female and 18 (15%) were male. The mean age of the patients was 49.4 ± 13.6 (range 21–83). Of the patients, 54 (44%) had received a kidney transplant from deceased donors and 69 (56%) had received a kidney transplant from living donors. The mean age of patients without bacterial growth in urine cultures was 46.8 ± 12.6, and 36.5% were male.

The most prevalent bacterial species identified were *Escherichia coli* (*E. coli*) 176 strains (49%) and *Klebsiella* spp. 88 strains (24%), together accounting for nearly three-quarters of all positive cultures. These findings highlight the predominance of Gram-negative bacteria in this patient population. Detailed distributions of the isolated organisms are presented in Table 1. Among seven *Staphylococcus* isolates, two were identified as *Staphylococcus aureus* and five as *coagulase-negative staphylococci* (*CoNS*). *S. aureus* bacteriuria was primarily observed in catheterized patients and considered clinically significant, whereas *CoNS* isolates were interpreted as possible contaminants.

Regarding antimicrobial resistance, *E. coli* isolates showed the highest resistance to ampicillin (64%) and ciprofloxacin (53%) while maintaining relatively low resistance to amikacin (7%) and carbapenems (≤5%). In *Klebsiella* spp., resistance was notably higher, particularly to ceftriaxone (52%), ciprofloxacin (62%), and trimethoprim–sulfamethoxazole (TMP-SMX) (60%), with moderate resistance to carbapenems (up to 28%). Detailed resistance rates, as well as extended-spectrum β-lactamase (ESBL) and carbapenemase (KPC) production, are summarized in Table 2.

When stratified by gender, *E. coli* remained the most frequently isolated pathogen in both groups but was more common among female recipients, whereas *Klebsiella* spp. and *Enterococcus* spp. were relatively more frequent in males (*p* = 0.006). Among male patients, *E. coli* isolates exhibited higher resistance to TMP-SMX (*p* = 0.019), while no gender-related differences were observed for other antibiotics. In *Klebsiella* isolates from males, resistance was higher to ciprofloxacin (*p* = 0.004), ertapenem (*p* = 0.002), gentamicin (*p* < 0.001), and TMP–SMX (*p* = 0.003). No significant gender-related differences were detected for *Enterococcus* isolates. Detailed counts and percentages are provided in Table 3.

When stratified by donor type (deceased vs. living-donor recipients), *Klebsiella* spp. were more frequent among deceased recipients, whereas *E. coli* predominated in living-donor recipients (*p* < 0.001). Overall antibiotic resistance rates did not differ between donor groups. The prevalence of *Enterococcus* spp. was similar across donor types; however, *Enterococcus* isolates from deceased-donor recipients showed higher ciprofloxacin resistance (*p* = 0.039). Detailed distributions are provided in Table 4.

In the age-stratified analysis, *E. coli* isolates from patients > 50 years showed higher resistance to TMP-SMX, reaching statistical significance (*p* = 0.05). No age-related differences were observed for other antibiotics. Moreover, no meaningful age-related disparities were noted in the distribution or resistance profiles of *Klebsiella* spp. or *Enterococcus* spp. Detailed counts and percentages are provided in Table 5.

The resistance rate of uropathogens isolated within the first year post-transplantation was found to be higher (Table 6). In *E. coli* isolates, resistance to gentamicin was 42% during the first year compared to 14% thereafter (*p* = 0.003), and resistance to TMP–SMX was 92% versus 42% (*p* < 0.01). In *Klebsiella* spp., resistance to ciprofloxacin (94% vs. 56%; *p* = 0.010), gentamicin (63% vs. 1%; *p* < 0.01), and TMP–SMX (100% vs. 51%; *p* < 0.01) was significantly higher in the first year after transplantation.

Over the two-year period, 37 patients experienced a single UTI episode, whereas 86 had recurrent infections (2–13 episodes). Given the small size of the single-infection group (*n* = 35), percentages are potentially unstable and should be interpreted with caution; absolute counts are reported in Table 7. In recurrent infections, *E. coli* isolates showed higher resistance to gentamicin (20% vs. 0%; *p* = 0.027), and *Klebsiella* spp. isolates showed higher resistance to TMP–SMX (63% vs. 10%; *p* = 0.010). No other between-group differences reached statistical significance.

**Figure 1:**
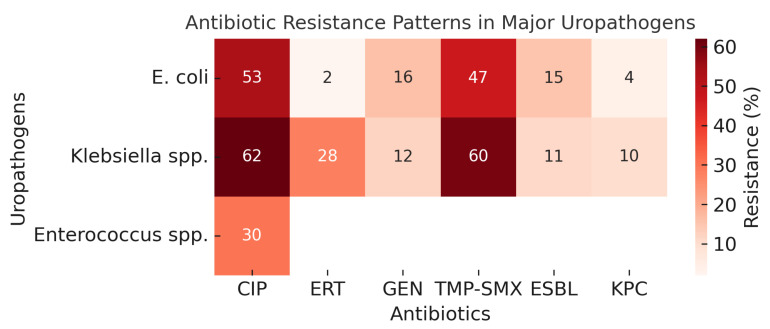
Heatmap illustrating antibiotic resistance rates among the most common uropathogens isolated from kidney transplant recipients. The heatmap shows resistance percentages for *E. coli*, *Klebsiella* spp., and *Enterococcus* spp. against six commonly tested antibiotics: ciprofloxacin (CIP), ertapenem (ERT), gentamicin (GEN), trimethoprim–sulfamethoxazole (TMP–SMX), extended-spectrum β-lactamase (ESBL), and carbapenemase (KPC) production. Warmer colors represent higher resistance rates. Klebsiella spp. demonstrated the highest resistance to ciprofloxacin and TMP–SMX, while carbapenem resistance remained relatively low across species.

Overall, the analysis revealed several clinically relevant resistance patterns. Ciprofloxacin and TMP–SMX resistance rates were high in both *E. coli* and *Klebsiella* spp. isolates. Resistance was notably higher in male recipients, during the first year post-transplantation, and in recurrent urinary infections. These findings underscore that resistance patterns in kidney transplant recipients are influenced not only by pathogen type but also by host and temporal factors, which should be taken into account in empirical treatment strategies (Figure 1).

## 3. Discussion

Antimicrobial resistance represents one of the most critical global health challenges and poses a particular threat to solid organ transplant recipients [6]. Immunosuppressive therapy, which is essential for preventing acute and chronic rejection, predisposes these patients to severe infectious complications. In addition, surgical manipulation of the urinary tract, altered anatomy, and the presence of urinary catheters further increase susceptibility to infection [7]. Antibiotic prophylaxis remains a cornerstone of infection prevention after kidney transplantation; however, inappropriate empirical therapy and prolonged antibiotic exposure have contributed to the emergence of resistant pathogens [8]. The kidney is the most frequently transplanted solid organ, and UTIs represent the most common post-transplant infection. Because microbial epidemiology and antibiotic susceptibility vary considerably over time and between regions, continuous local surveillance is crucial to guide empirical therapy [7]. Therefore, this study investigated the distribution of uropathogens and their antibiotic resistance profiles among kidney transplant recipients, with particular attention to temporal variations during the early and late post-transplant periods.

During the study period (2023–2024), a total of 363 episodes of significant bacteriuria were identified among 123 kidney transplant recipients. The overall bacterial growth rate of 18% observed in urine cultures obtained from the kidney transplant service was comparable to the findings of Korth et al. [9], yet notably lower than the 32% reported by Shapouri Moghaddam et al. [8]. These variations may be attributable to differences in patient follow-up duration, diagnostic thresholds for significant bacteriuria, or regional infection control practices.

In this study, *Escherichia coli*, *Klebsiella* spp. and *Enterococcus* spp. were identified as the predominant uropathogens among kidney transplant recipients, consistent with previous national and international studies [7,8,9]. A recent report from our country similarly found *E. coli* (59%), *Klebsiella* spp. (17%), and *Enterococcus* spp. (7%) as the most frequent causative agents [10], closely aligning with our findings. However, some studies have documented markedly higher *Enterococcus* isolation rates (24–40%), particularly in cohorts with prolonged hospitalization and frequent catheter use [7,11,12,13]. The predominance of *E. coli* can be attributed to its strong uropathogenic virulence factors and ability to colonize the urinary tract, whereas *Enterococcus* infections are often associated with invasive devices and prior antimicrobial exposure. Consistent with previous evidence, coagulase-negative staphylococci were generally considered contaminants, whereas *Staphylococcus aureus* isolates were regarded as clinically significant, especially in catheterized patients [14].

In the present study, resistance among *E. coli* isolates was highest to ciprofloxacin (53%) and TMP–SMX (47%), with lower resistance to ceftriaxone (37%), gentamicin (16%), and ertapenem (2%). These findings are comparable to national data showing ciprofloxacin resistance of 60%, ceftriaxone 41%, gentamicin 37%, and TMP–SMX 86% [15], and align with international reports describing 50% ciprofloxacin and 38–100% TMP–SMX resistance [7,9,16,17]. For *Klebsiella* spp., the resistance rates were 62% to ciprofloxacin, 52% to ceftriaxone, 28% to ertapenem, 12% to gentamicin, and 60% to TMP–SMX, mirroring European data where *Klebsiella* species are generally more resistant than *E. coli* [7,9]. Extended-spectrum β-lactamase (ESBL) production, a key resistance mechanism among Enterobacteriaceae, was detected in 15% of *E. coli* and 11% of *Klebsiella* isolates. The reported ESBL positivity rates in the literature range from 23% to 52% [6,15]. The relatively low ESBL rates observed in our center may be attributable to the institutional antibiotic stewardship policy, which includes a single perioperative dose of ceftriaxone (2 g IV) and routine TMP-SMX prophylaxis (80/400 mg once daily for at least six months). This regimen aligns with current European and is in line with the Kidney Disease: Improving Global Outcomes (KDIGO) recommendations advocating limited perioperative antibiotic exposure and standardized post-transplant prophylaxis to reduce resistance selection pressure [18,19]. In contrast, centers using extended perioperative regimens have reported higher ESBL rates (20–40%). Therefore, adherence to concise prophylaxis protocols and rational antibiotic policies may help maintain lower resistance levels in transplant populations [20]. Among *Enterococcus* isolates, 28% showed resistance to ciprofloxacin, while all remained susceptible to vancomycin, linezolid, and teicoplanin. In contrast, Samanipour et al. reported higher rates of vancomycin-resistant *Enterococcus* (VRE) in kidney transplant units, which they attributed to the frequent use of vancomycin for hemodialysis catheter-associated infections [16].

In this study, the majority of kidney transplant recipients with UTIs were female (85%). This finding is consistent with previous studies reporting a higher UTI incidence among women, which is largely attributed to anatomical and physiological factors such as a shorter urethra and the proximity of the urethral meatus to the perineal flora [10,12]. In our study, *E. coli* was more frequently isolated from female patients, whereas *Klebsiella* and *Enterococcus* species were predominant in males, suggesting that gender may influence pathogen distribution. Interestingly, male patients exhibited significantly higher antimicrobial resistance rates, consistent with the observations of Rostkowska et al. [7]. This difference may be related to the higher prevalence of complicated UTIs in men, often associated with prostatitis, urinary outflow obstruction, or long-term catheterization. These conditions promote bacterial persistence, biofilm formation, and repeated antibiotic exposure—factors that collectively contribute to the selection of resistant strains [21]. Consequently, the management of male transplant recipients should incorporate these gender-specific risk factors, emphasizing targeted empirical therapy and close microbiological monitoring.

In our study, *Klebsiella* spp. isolates were more frequently recovered from recipients of deceased donor kidneys, whereas *E. coli* isolates predominated in those receiving grafts from living donors. This distinction suggests that donor-related factors and peri-transplant conditions may influence the post-transplant microbiological spectrum. Deceased donor transplants are often associated with longer ischemia times, intensive care exposure, and broad-spectrum antibiotic use prior to organ procurement—all of which can promote colonization or transmission of opportunistic pathogens such as *Klebsiella* spp. In contrast, living donor transplants typically involve shorter hospitalization periods and fewer perioperative interventions, which may favor infections caused by community-acquired pathogens such as *E. coli*. Although our analysis did not reveal statistically significant differences in antibiotic resistance between donor types, other studies have demonstrated that infections associated with deceased donor transplants tend to involve more resistant organisms [12,22]. These findings underscore the importance of tailoring infection prevention strategies and empirical antibiotic choices according to donor characteristics and perioperative clinical context.

Older kidney transplant recipients are inherently more susceptible to UTIs due to age-related physiological changes, decreased immune responsiveness, and higher rates of comorbidities. Previous studies have also reported that antimicrobial resistance among uropathogens tends to increase with patient age in transplant populations [7,12,22]. In our study, the mean age of patients with positive urine cultures was higher than that of those without bacterial growth, and *E. coli* isolates from patients over 50 years of age demonstrated significantly greater resistance to TMP-SMX. This may reflect the cumulative effect of repeated antibiotic exposure, chronic colonization, and reduced renal clearance in older individuals, all of which can contribute to resistance selection. By contrast, age-related differences were not significant for *Klebsiella* and *Enterococcus* species, suggesting that host factors may influence *E. coli* dynamics more strongly than other pathogens.

The highest resistance rates were observed within the first year following kidney transplantation. Notably, TMP–SMX resistance reached very high levels in both *E. coli* and *Klebsiella* isolates during this period, suggesting that the continued effectiveness of TMP–SMX prophylaxis in early post-transplant management warrants re-evaluation. Several cross-sectional studies have similarly focused on early post-transplant infections, reporting elevated resistance during the first year when immunosuppression and antibiotic exposure are most intense [2,17,22]. The pronounced resistance in this phase likely reflects selective pressure from prophylactic regimens, cumulative antimicrobial exposure, and the transient immunological vulnerability characteristic of the early post-transplant period. To the best of our knowledge, there is limited evidence addressing resistance trends beyond the first year after transplantation. Longitudinal surveillance of late post-transplant infections is therefore essential to guide duration and choice of prophylactic therapy in this high-risk population.

Recurrent UTIs are a major clinical concern in kidney transplant recipients and are often associated with increased antimicrobial resistance [9,23]. In our study, resistance rates were significantly higher in recurrent episodes than in single infections. This trend may reflect the cumulative impact of repeated hospitalizations, prolonged or empirical antibiotic exposure, and persistence of uropathogenic strains capable of biofilm formation. Such factors facilitate bacterial survival despite treatment, promoting the emergence of multidrug-resistant clones. Notably, high ciprofloxacin resistance likely results from its widespread use as empirical therapy for complicated UTIs in both inpatient and outpatient settings. Similarly, the routine administration of TMP–SMX for *Pneumocystis jirovecii* prophylaxis and UTI prevention may have contributed to the elevated TMP–SMX resistance observed in our study and others [16,24]. These findings suggest that although TMP–SMX remains a guideline-recommended prophylactic agent, its diminishing efficacy against urinary pathogens warrants careful local evaluation and periodic adjustment of prophylactic regimens based on resistance surveillance.

This study has several limitations. First, the relatively small denominator in the single-infection subgroup (*n* = 35) may have produced imprecise percentage estimates and limited the robustness of between-group comparisons. Second, multiple UTI episodes per patient were analyzed as independent events. Because of the modest sample size (123 patients and 363 episodes), the application of mixed-effect or generalized estimating equation (GEE) models to account for repeated measures was not feasible, as such modeling would likely yield unstable estimates. Effect size measures such as odds ratios and confidence intervals were also not calculated due to small and uneven subgroup sizes; therefore, the reported *p*-values should be interpreted as indicators of statistical association rather than precise effect magnitude. Moreover, data on prior antibiotic exposures were not collected in sufficient detail to assess their potential influence on resistance patterns. Finally, this was a single-center study, and the findings may not be generalizable to all kidney transplant populations. Future multicenter, prospective studies with larger and more diverse cohorts—including longitudinal antibiotic exposure data—are warranted to validate these findings and provide a broader understanding of antimicrobial resistance dynamics in transplant recipients.

In conclusion, this study provides a detailed overview of uropathogen distribution and antibiotic resistance among kidney transplant recipients at our center. Despite adherence to rational antibiotic use policies, the resistance rates—particularly to ciprofloxacin and TMP-SMX—remain markedly high. These findings suggest that both agents may no longer represent suitable empirical options for the initial management of urinary tract infections in this population. Our results also highlight clinically relevant trends, including higher resistance in male patients and during the first post-transplant year, underscoring the importance of individualized, risk-based antibiotic selection. Empirical and prophylactic regimens should be guided by continuous local resistance surveillance and reinforced by routine culture-based confirmation of pathogens and susceptibilities. Given the regional variability in uropathogens and their resistance patterns, periodic multicenter evaluations are essential to inform antibiotic stewardship and optimize outcomes for kidney transplant recipients.

## 4. Materials and Methods

### 4.1. Study Design and Population

At Bursa Uludağ University Hospital, a 900-bed tertiary care hospital in Turkey, adult (aged ≥ 18 years) patients with a history of kidney transplantation who were hospitalized in the Nephrology Organ Transplantation Clinic between 1 January 2023, and 31 December 2024, and who had growth in their urine culture were retrospectively reviewed. The microorganisms and susceptibility patterns were obtained from the EpiCenter™ (Becton Dickinson, Franklin Lakes, NJ, USA) data management system. Urine cultures that exhibited repeated growth of the same microorganism within a three-week period were excluded from this study. Furthermore, demographic data including the patient’s age, gender, transplant date, and donor type (living/deceased) were retrieved from the hospital information system (MIA MED, Akdeniz, Turkey). Catheterization referred to temporary urinary devices used in the early postoperative period, including Foley catheters and ureteral stents, which were generally removed within 7–14 days. A few patients required indwelling catheters or nephrostomy tubes due to post-transplant complications; however, detailed data on device duration were not available. Perioperative antibiotic prophylaxis consisted of a single preoperative dose of ceftriaxone (2 g IV) administered within 60 min before incision. Postoperative antibiotics were not routinely continued unless clinically indicated. All kidney transplant recipients received oral TMP-SMX (80/400 mg once daily) for at least six months post-transplantation for prophylaxis against Pneumocystis jirovecii pneumonia and urinary tract infections.

### 4.2. Identification and Antimicrobial Susceptibility Testing

Urine samples from patients were inoculated onto blood agar and Eosin Methylene Blue (EMB) agar plates (BD, Heidelberg, Germany) and incubated at 35 °C for 24–48 h. The evaluation was based on the Clinical Microbiology Specialists Association (KLİMUD) Urine Guide from Sample to Result, and bacterial growth of 10^5^ colony-forming units (CFU)/mL in the urine culture was considered significant [25]. The causative microorganisms were identified through the use of matrix-assisted laser desorption ionization time-of-flight mass spectrometry (MALDI-TOF MS) (Bruker Daltonik GmbH, Bremen, Germany). Antibiotic susceptibility testing was performed using the Phoenix ID System (Becton Dickinson Diagnostics, Franklin Lakes, NJ, USA) and evaluated according to the recommendations of the European Committee on Antimicrobial Susceptibility Testing (EUCAST v13.0, 2023) criteria [26]. The antibiotic panels tested varied by organism group as follows: For *Enterobacterales* (including *E. coli* and *Klebsiella* spp.): ampicillin, ceftriaxone, ciprofloxacin, gentamicin, amikacin, piperacillin–tazobactam, ertapenem, imipenem, fosfomycin, nitrofurantoin, and TMP-SMX. For *Enterococcus* spp.: ampicillin, vancomycin, and linezolid. For *Pseudomonas aeruginosa* and *Acinetobacter* spp.: ciprofloxacin, gentamicin, amikacin, imipenem, and piperacillin–tazobactam. For *Staphylococcus aureus* and *coagulase-negative staphylococci* (*CoNS*): oxacillin, cefoxitin, ciprofloxacin, gentamicin, and linezolid. Extended-spectrum β-lactamase (ESBL) production was confirmed phenotypically using the combination disk method with cefotaxime and ceftazidime disks, with and without clavulanic acid, according to EUCAST recommendations. Carbapenemase activity was screened by the modified Hodge test and confirmed using inhibitor-based assays (including boronic acid and EDTA synergy tests) for the detection of KPC and OXA-48 enzymes. Molecular testing was not routinely performed. Although a broader antibiotic panel was tested according to EUCAST guidelines, only clinically relevant and representative agents were included in the tables and analyses to ensure clarity and focus on the most significant resistance trends. Percentages of resistance were calculated based on the number of isolates tested for each antibiotic (n/N). According to EUCAST interpretive categories, isolates were classified as susceptible (S), susceptible, increased exposure (I), or resistant (R). For descriptive analysis, the S and I categories were combined and reported together as “susceptible,” following EUCAST reporting recommendations. For internal control, *Staphylococcus aureus* ATCC^®^ 29213™, *Escherichia coli* ATCC^®^ 25922™, and *Pseudomonas aeruginosa* ATCC^®^ 27853™ strains were used.

### 4.3. Statistical Analysis

Statistical analyses were performed using SPSS version 28.0 (IBM Corp., Armonk, NY, USA). *p* < 0.05 was considered statistically significant. Descriptive data were presented as numbers and percentages, while continuous variables were expressed as mean ± standard deviation or median (minimum–maximum). Categorical variables were compared between independent groups. Comparisons between groups were performed using the chi-squared test and Fisher’s exact test when necessary. A *p*-value of <0.05 was considered statistically significant.

## 5. Conclusions

This study highlights the distribution of uropathogens and antibiotic resistance among kidney transplant recipients at our center. Despite rational antibiotic use policies, resistance—particularly to ciprofloxacin and TMP-SMX—remains high. These findings suggest that both agents may no longer be optimal empirical options for urinary tract infections in this population. Continuous local resistance surveillance and culture-based therapy are essential to guide empirical treatment and optimize outcomes in kidney transplant recipients.

## Figures and Tables

**Table 1:** Distribution of Uropathogens Detected in Kidney Transplant Patients.

Uropathogens	Number of Strains	Percent (%)
*Escherichia coli*	176	49
*Klebsiella* spp.	88	24
*Enterococcus* spp.	23	6
*Streptococcus agalactiae*	9	2
*Staphylococcus* spp. ^1^	7	2
*Enterobacter* spp.	7	2
*Proteus* spp.	6	2
*Acinetobacter* spp.	6	2
*Pseudomonas aeruginosa*	4	1
*Candida* spp.	12	3
Others ^2^	25	7
Total	363	100

^1^: Two were identified as Staphylococcus aureus and five as *coagulase-negative staphylococci* (*CoNS*). ^2^: Non–group B *Streptococcus* spp. (*n* = 6), *Chryseobacterium* spp. (*n* = 2), *Citrobacter freundii* (*n* = 4), *Moraxella catarrhalis* (*n* = 3), *Morganella morganii* (*n* = 4), *Serratia marcescens* (*n* = 3), *Ureaplasma urealyticum* (*n* = 2), and *Stenotrophomonas maltophilia* (*n* = 1); total *n* = 25. Low-frequency taxa not shown separately were grouped under Others to preserve table readability.

**Table 2:** Antimicrobial Resistance Rates of Uropathogens in Kidney Transplant Patients.

Uropathogens	CIP (%)	ERT (%)	GEN (%)	TMP-SMX (%)	ESBL (%)	KPC (%)
Gram-negative bacteria						
*E. coli* (*n* = 176)	53	2	16	47	15	4
*Klebsiella* spp. (*n* = 88)	62	28	12	60	11	10
*Enterobacter* spp. (*n* = 7)	29	14	0	29	-	-
*Proteus* spp. (*n* = 6)	67	0	50	83	-	-
*Acinetobacter* spp. (*n* = 6)	100	100	67	67	-	-
*P. aeruginosa* (*n* = 4)	75	100	-	-	-	-
Gram-positive bacteria						
*Enterococcus* spp. (*n* = 23)	30	-	-	-	-	-
*Staphylococcus* spp. (*n* = 9)	71	-	14	29	-	-

Abbreviations: CIP, ciprofloxacin; ERT, ertapenem; GEN, gentamicin; TMP–SMX, trimethoprim–sulfamethoxazole; ESBL, extended-spectrum β-lactamase; KPC, *Klebsiella pneumoniae* carbapenemase. Formatting note: Values are expressed as percentages of isolates (one decimal place). “-“ indicates not tested or not available. Note: Percentages reflect only the isolates tested for each antibiotic (n/N).

**Table 3:** Distribution of Uropathogens and Antimicrobial Resistance Rates by Gender.

	Uropathogens	Rate (%)	CIP (%)	ERT (%)	GEN (%)	TMP-SMX (%)	ESBL (%)	KPC (%)
Male (*n* = 58)	*E. coli* (*n* = 20)	35	75	0	20	75	5	0
	*Klebsiella* spp. (*n* = 18)	31	94	61	50	94	17	17
	*Enterococcus* spp. (*n* = 8)	25	29					
Female (*n* = 305)	*E. coli* (*n* = 160)	52	51	3	18	44	16	4
	*Klebsiella* spp. (*n = 69*)	23	54	20	3	52	10	9
	*Enterococcus* spp. (*n* = 16)	5	31					

**Table 4:** Distribution of Uropathogens and Antimicrobial Resistance Rates by Donor Type.

	Uropathogens	Rate (%)	CIP (%)	ERT (%)	GEN (%)	TMP-SMX (%)	ESBL (%)	KPC (%)
Deceased Donor (*n* = 154)	*E. coli* (*n* = 64)	42	56	3	19	52	17	2
	*Klebsiella* spp. (*n* = 54)	35	70	28	17	67	9	9
	*Enterococcus* spp. (*n* = 10)	7	60					
Living Donor (*n* = 209)	*E. coli* (*n* = 115)	55	53	2	17	46	13	5
	*Klebsiella* spp. (*n* = 34)	16	50	29	6	50	15	12
	*Enterococcus* spp. (*n* = 13)	6	15					

**Table 5:** Distribution of Uropathogens and Antimicrobial Resistance Rates by Patient Age.

	Uropathogens	Rate (%)	CIP (%)	ERT (%)	GEN (%)	TMP-SMX (%)	ESBL (%)	KPC (%)
≤50 age (*n* = 196)	*E. coli* (*n* = 100)	51	56	3	13	38	14	5
	*Klebsiella* spp. (*n* = 49)	25	65	33	6	63	14	14
	*Enterococcus* spp. (*n* = 13)	7	31					
>50 age (*n* = 166)	*E. coli* (*n* = 80)	48	53	1	24	60	15	3
	*Klebsiella* spp. (*n* = 39)	24	59	23	21	56	8	5
	*Enterococcus* spp. (*n* = 10)	6	40					

**Table 6:** Uropathogen Distribution and Antimicrobial Resistance Rates by Post-transplantation Time.

	Uropathogens	Rate (%)	CIP (%)	ERT (%)	GEN (%)	TMP-SMX (%)	ESBL (%)	KPC (%)
First year (*n* = 58)	*E. coli* (*n* = 24)	41	63	8	42	92	13	8
	*Klebsiella* spp. (*n* = 16)	28	94	44	63	100	13	6
	*Enterococcus* spp. (*n* = 7)	12	43					
2–28 years (*n* = 301)	*E. coli* (*n* = 155)	52	54	1	14	42	8	2
	*Klebsiella* spp. (*n* = 72)	24	56	25	1	51	11	11
	*Enterococcus* spp. (*n* = 16)	5	27					

**Table 7:** Comparison of Resistance Ratios in Single and Recurrent Infections.

	Uropathogens	Rate (%)	CIP (%)	ERT (%)	GEN (%)	TMP-SMX (%)	ESBL (%)	KPC (%)
Single infections (*n* = 35)	*E. coli* (*n* = 20)	57	35	5	0	30	25	10
	*Klebsiella* spp. (*n* = 5)	14	40	40	0	10	20	20
	*Enterococcus* spp. (*n* = 3)	9	0					
Recurrent infections (*n* = 327)	*E. coli* (*n* = 160)	49	57	2	20	50	13	3
	*Klebsiella* spp. (*n* = 83)	25	64	28	13	63	11	10
	*Enterococcus* spp. (*n* = 20)	6	37					

## Data Availability

Anonymized data are available on request.

## Abbreviations

The following abbreviations are used in this manuscript:

UTI	Urinary tract infection
TMP-SMX	trimethoprim-sulfamethoxazole
ESBL	Extended spectrum beta lactamase
KPC	Carbapenemase
